# Monitoring caesarean births using the Robson ten group classification system: A cross-sectional survey of private for-profit facilities in urban Bangladesh

**DOI:** 10.1371/journal.pone.0220693

**Published:** 2019-08-08

**Authors:** Tahmina Begum, Herfina Nababan, Aminur Rahman, Md Rajibul Islam, Alayne Adams, Iqbal Anwar

**Affiliations:** 1 Health Systems and Population Studies Division, icddr,b, Dhaka, Bangladesh; 2 The Institute for Social Science Research, The University of Queensland, Brisbane, Australia; 3 Nossal Institute for Global Health, School of Population and Global Health, the University of Melbourne, Melbourne, Australia; 4 Department of International Health, Georgetown University, Washington, United States of America; 5 James P Grant School of Public Health, Dhaka, Bangladesh; Aga Khan University, KENYA

## Abstract

**Background:**

Globally, Caesarean section (CS) rates are mounting and currently exceed the safe upper limit of 15%. Monitoring CS rates using clinical indications and obstetric sub-group analysis could confirm that women in need have been served. In Bangladesh, the reported CS rate was 31% in 2016, and almost twice that rate in urban settings. Delivering in the private healthcare sector was a strong determinant. This study uses Robson Ten Group Classification System (TGCS) to report CS rates in urban Bangladesh. The clinical causes and determining factors for CS births have also been examined.

**Methods:**

This record linkage cross-sectional survey was undertaken in 34 urban for-profit private hospitals having CS facilities during the period June to August 2015. Data were supplied by inpatient case records and operation theatre registers. Descriptive analyses were performed to calculate the relative size of each group; the group-specific CS rate, and group contribution to total CS and overall CS rate. CS indications were grouped into eleven categories using ICD 10 codes. Binary logistic regression was performed to explore the determinants of CS.

**Results:**

Out of 1307 births, delivery by CS occurred in 1077 (82%). Three obstetric groups contributed the most to overall CS rate: previous CS (24%), preterm (23%) and term elective groups (22%). The major clinical indications for CS were previous CS (35%), prolonged and obstructed labor (15%), fetal distress (11%) and amniotic fluid disorder (11%). Multiple gestation, non-cephalic presentation, previous bad obstetric history were positive predictors while oxytocin used for labour induction and increased parity were negative predictors of CS.

**Conclusions:**

As the first ever study in urban private for-profit health facilities in Bangladesh, this study usefully identifies the burden of CS and where to intervene. Engagement of multiple stakeholders including the private sector is crucial in planning effective strategies for safe reduction of CS.

## Introduction

According to the World Health Organization (WHO), the population-based Caesarean Section (CS) rate should range between 10–15%[1] to positively impact maternal and neonatal health outcomes[2, 3]. However, recent data from 150 countries indicate that average CS rate is 19%, ranging from a high of 40% in Latin America to a low of 7% in the African region[4]. This socio economic disparity in CS service utilization has raised questions about whether the right women are receiving the right service at the right time[5, 6]. To answer this question, the population level CS rate is too crude to be useful[7] as clinical indications for CS data are missing. Monitoring and evaluation data on CS indications are also problematic as they are collected retrospectively and do not identify particular obstetric risk groups[8, 9]. It is also the case that CS rates may vary between health facilities depending on the obstetric risk groups they serve.

In summary, the CS rate of any particular labor unit can only be considered appropriate when the required obstetric information is available to justify medical indications for the procedure[5]. In this context, a common classification system that transforms crude data into useful information is required to analyze and compare CS rates at local, national and international levels in a consistent manner[5]. Accordingly, the Robson Ten Group Classification System (TGCS) has emerged. It is recognized as a highly effective monitoring tool that provides optimum maternal and fetal epidemiological information[10], and is well-supported by meta-analysis findings[11].

The Robson TGCS uses basic obstetric characteristics like parity, previous CS, gestational weeks, type of labor onset, presentation and number of fetuses to classify birthing women into ten different groups[8]. Each group is totally inclusive but mutually exclusive without any obstetric risk adjustment[8]. The TGCS is simple and easy to implement even in low income settings. It can also be used to identify group-specific CS rates and enable focused intervention given that management of labor varies between groups[8, 12]. WHO, in their 2015 Executive Statement on CS, recommended that Robson TGCS be at the health facility and national levels for the monitoring and evaluation of CS rates[7]. If employed in this manner, and good clinical practice is maintained, a reduction in population level CS rate is possible.[1]

National maternal mortality survey data from Bangladesh has documented the CS rate as 31% in 2016[13], with significant variation across the country and between private and public sectors [14].The CS rate for urban women was double (36%) that of rural women (18%)[14], and delivering in private for-profit health facilities was identified as the strongest determinant[15, 16]. Financial incentives were reported as the main driver for CS births in private facilities, instead of established clinical risks [17–20]. However, the autonomous nature of the private sector has limited a more nuanced understanding of CS related patterns and practices. To fill this knowledge gap, our study explores CS rates among selected private for-profit health facilities in urban Bangladesh using Robson TGCS. Study results will be useful in formulating obstetric group-specific intervention strategies to maintain an optimum CS rate, and will provide a benchmark for future comparison across a variety of hospitals and regions in Bangladesh.

## Materials & methods

### Study design and setting

This record linkage health facility-based cross-sectional survey was conducted in Sylhet City Corporation, Bangladesh in 2015. For-profit health hospitals providing CS services were located using icddr,b’s Urban Health Atlas [21]. A total of 34 private hospitals were identified with variable inpatient bed capacity: 18 hospitals with an <20 bed capacity; 13 with a 21 to 100 bed capacity and 3 medical college hospitals with a 450 to 1000 bed capacity. Study participants were pregnant women with more than 29 weeks’ gestation birth outcomes occurring in the period of 29^th^ May to 28^th^ August 2015.

#### Data source and sample size

Data for this manuscript are provided by a baseline survey for a pre-post intervention study undertaken in the aforementioned private health facilities. Additional data sources included inpatient case records and operation theatre register records. A structured checklist installed in a “digital tablet” was used to retrieve data from case records. The required number of case records to be reviewed was set based on the existing prevalence of key evidence-based techniques; and then adjusted upwards to account for any design effects. Five evidence-based clinical practices were considered: Active management of Third Stage of Labour (AMTSL); partograph use; birth companion present during labour; advice and offer of family planning services and advice on danger signs. The minimum required sample was 910 case records. The period of data collection was set to three months based on documented delivery patient turnover rates in the sample hospitals. During this period, a total of 1343 case records were identified and included in analysis, including at least 35 cases from each small and medium-sized private hospital in the sample, and 70 cases from each medical college hospital. CS indication data were retrieved from operation theatre registers if they were not available in the case record file.

### Statistical analysis

STATA 15 software was used for data analysis. The Robson TGCS is comprised of six major variables—parity, onset of labour (spontaneous or induced), gestational weeks, fetal presentation, number of fetuses, previous caesarean delivery. These variables are used to group delivered women into ten obstetric groups. Analysis of these groups considers the following measures: 1) ***“Relative size of the group”*** based on the number of women in each group divided by total number of women giving birth; 2) “***Group specific CS rate”*** which is the CS divided by the number of women in each group; 3) ***“Group contribution to total CS rate***” or the number of CS over the total number of women undergoing caesarean; and, 4) “**Group contribution to overall CS rate”** which is the number of CS over the total number of women giving birth. Given that this is the first study of CS in private health settings in Bangladesh, data quality was a concern[22]. For this reason, reference values from earlier studies were used to validate the relative size of the each Robson group [10, 12, 23].

Clinical indications for CS were also analyzed using the International Classification of Disease (ICD) 10 code version[24] both separately and for eleven different categories based on cumulative percentage rates.

The characteristics of study participants in terms of maternal and fetal parameters were explored using frequency percentages. Maternal characteristics were age, parity, previous Bad Obstetric Histories (BOH), and use of uterotonics. The BOH of the mother was dichotomized to “yes” or “no” if any of the following conditions was present in her earlier pregnancies including pregnancy induced hypertension, eclampsia, still birth, abortions, congenital anomaly of fetus, ante or post-partum haemorrhage, preterm delivery, cervical tear, perineal tear, manual removal of placenta, delivery by CS or any other relevant issue. Uterotonics refer to the use of oxytocin before delivery to initiate or augment labour pain. Other exposure variables were fetal presentation, number of gestations, birthweight, and fetal presentation. The number of gestations variable was categorized into two sub-categories; single and multiple, where multiple denotes twin and triplet pregnancies. Fetal presentation was categorized as cephalic or non-cephalic, inclusive of breech, transverse and oblique lie.

As appropriate, bivariate analysis using chi square or t-test were applied to test the statistical association between maternal and fetal characteristics and CS outcome at a p value of <0.05. Both significant and clinically relevant non-significant factors were included in logistic regression models using a backward elimination method. The prerequisite of logistic regression analysis, “exposure variables are independent of each other” was not applicable in this instance as the outcome of interest varied by hospital size (number of hospital beds). Thus, to minimize possible biases from intra-cluster correlation, we adjusted our logistic model for cluster effect [25]. Variables found significant at a 95% confidence interval according to the adjusted model were considered to have a statistically significant association with CS outcome.

### Ethics statement

Ethical approval to conduct this research was obtained from the Institutional Review Board (IRB) of icddr,b under protocol number 14107. The IRB is comprised of a Research Review Committee (RRC) and an Ethical Review Committee (ERC). The RRC considers the research merit and technical feasibility of the protocol. Whereas the ERC review aims to ensure privacy and anonymity of the study participants and that proper informed consent is obtained before reviewing patient records. Informed consent to access the patients’ record file was obtained from the respective private clinic and hospitals owners considering them as data custodian. Individual patient records were assigned a unique identification number, as was each of the study hospitals. Only de-identified data were used for analysis and reporting.

## Results

### Robson ten group classification and contribution to overall CS

Out of 1343 case records, 36 cases were discarded since essential variables required to initiate Robson TGCS classification were missing. The total sample included for analysis was 1307 birth events.

The overall CS rate was 82% ranging from a minimum rate of 50% to a maximum rate of 100%.

Table 1 describes the group-specific CS rate of women falling into each of the ten obstetric groups and their contribution to overall CS rate. The preterm group was the largest among women attending for childbirth (30%), followed by previous CS (24.6%), term elective (25%—both nuli and multi parous) and other groups (18%). The combined size of “Groups three & four” (Multiparous term) was appropriately higher (25%) than the cumulative percentages of “Groups one & two” (15.4%). Breech pregnancies comprising of “Groups six & seven” was 2.7%. All multiple pregnancies in “Group eight” and all abnormal lies in “Group nine” were well within the expected range, at 2.2% and 0.4% respectively. However, the “Group size 10” (preterm pregnancies) was higher (30%). However, the top three Robson groups contributing highly on overall CS rate were 24.6% for previous CS, 23% for preterm, and 22.1% for the term elective group.

**Table 1:** Robson ten obstetric groups and their contribution to overall Caesarean section (CS) rate.

	Robson Group	Number of women ^¥^n1	Number of CS ^£^n2	Relative size of group (%) (n1/N1)	Group specific CS (%) (n2/n1)	Group input to total CS (%) (n2/N2)	Group input to overall CS (%) (n2/N1)
1	Nulliparous, single cephalic, ≥37 weeks spontaneous labor	80	53	6.1	66	4.9	4.1
2	Nulliparous, single cephalic, ≥37 weeks, induced or pre-labour CS	122	121	9.3	99	11.2	9.3
2a	Nulliparous, single cephalic, ≥37 weeks induced labour	22	21	1.7	95	1.9	1.6
2b	Nulliparous, single cephalic, ≥37 weeks, pre-labor CS	100	100	7.7	100	9.3	7.7
3	Multiparous, single cephalic, ≥37 weeks, spontaneous labour (excluding previous CS)	158	48	12.1	30	4.5	3.7
4	Multiparous, single cephalic, ≥37 weeks, induced or pre-labour CS (exclude previous CS)	167	167	12.8	100	15.5	12.8
4a	Multiparous, single cephalic, ≥37 weeks, induced labour (excluding previous CS)	24	24	1.8	100	2.2	1.8
4b	Multiparous, single cephalic, ≥37 weeks pre-labour CS (excluding previous CS)	143	143	10.9	100	13.3	10.9
5	Previous CS, single cephalic, ≥37 weeks	322	322	24.6	100	29.9	24.6
6	All nulliparous breeches	16	16	1.2	100	29.9	1.2
7	All multiparous breeches (including previous CS)	19	17	1.5	89	1.6	1.3
8	All multiple pregnancies (including previous CS)	29	27	2.2	93	2.5	2.1
9	All transverse oroblique lies (including previous CS)	5	5	0.4	100	0.5	0.4
10	All preterm single cephalic, <37 weeks, (including previous CS)	389	301	30	77	27.9	23.0
	**Sub-total**	^©^N1 **= 1307**	^®^N2 **= 1077**				N2/N1 **= 82%**
	**Unclassified**	**36**	**0**				
	**Total**	**1343**	**1077**				**80%**

CS = Caesarean Section;

^©^N1 = Total births included in analysis;

^®^N2 = Total CS births included in analysis;

^¥^n1 = number of women in each Robson group;

^£^n2 = number of CS births in each Robson group;

**Fig 1:**
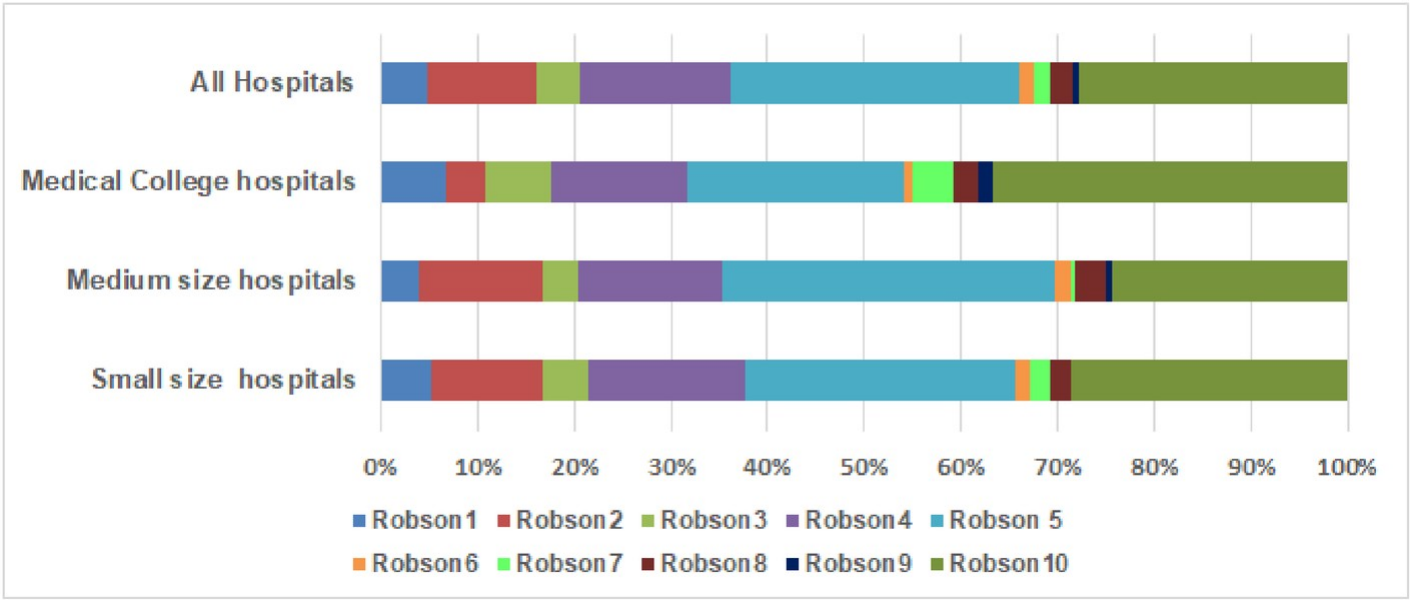
Contribution of each Robson group to overall CS rates by type of hospital in urban Bangladesh.

Fig 1 presents variations in CS rate across the Robson TGCS by type of hospital. The major driver of CS among large medical college hospitals was “Robson 10”, the preterm group (37%), while for medium-sized hospitals it was “Robson five” representing previous CS (34%), and for small-sized hospitals an almost equal proportion of previous CS (28%) and preterm CS (29%) was apparent. CS percentages for “Robson nine”, which includes abnormal presentation were high in medical college hospitals and nil in small hospitals.

### Clinical indications of CS

**Fig 2:**
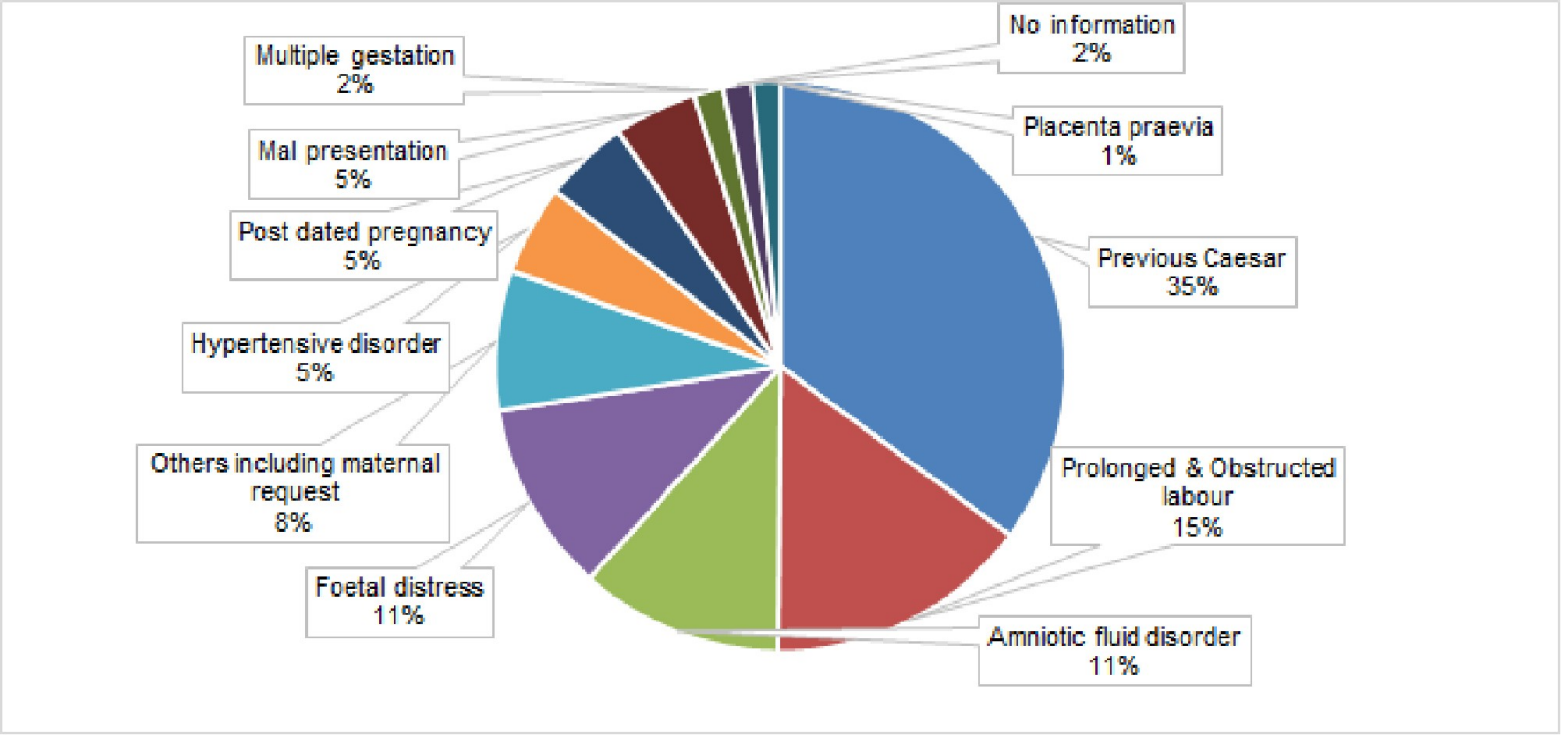
Clinical indications of CS in for-profit private hospitals in urban Bangladesh.

Clinical indications for CS were grouped into eleven different categories: hypertensive disorder (include gestational hypertension, eclampsia, preclampsia, unspecified hypertension); malpresentation (includes cephalopelvic disproportion, breech and transverse lie); disorder of amniotic fluid (covers both oligo and poly hydramnios); placenta praevia; post-dated pregnancy; prolonged and obstructed labour; fetal distress (isolated fetal distress or fetal distress associated with meconium stained amniotic fluid); previous CS; multiple pregnancies (twin and triplet), and “other” reported CS indications including general diseases complicating pregnancy (anaemia, asthma, diabetes, HBs Ag positive, Rh-ve mother), maternal request, and full term pregnancy with labor pain. The most commonly reported CS indication was repeat CS (35%), prolonged and obstructed labor (15%), fetal distress and amniotic fluid disorder 11% each (Fig 2).

Maternal characteristics, segregated by mode of childbirth, are presented in Table 2. The largest proportion of mothers giving birth (normally and by CS) were in their twenties, and less than 10% were teenagers. More than half of mothers were multiparous (having more than one child) and over 50% gave birth at 37 completed gestational weeks. Almost all gestations were singletons with cephalic presentation. However, for the large majority of women (over 80%), no information indicating bad obstetric history was available and most commonly reported previous bad obstetric history was previous CS. About 63% of women were identified as having a first time CS, whereas the percentage of normal vaginal births after prior CS was very low (1.7%). (data not shown) In terms of birth outcomes, the majority of newborns in both groups had normal birth weight, and only small percentage of pregnancies resulted in still birth.

**Table 2:** Characteristics of study respondents by mode of delivery in for-profit private hospitals in urban Bangladesh.

	Normal delivery n (%)	CS n (%)	P value
	N = 230	N = 1077	
**Maternal Age (year)**			
≤19	18 (7.9)	61 (5.7)	0.325
20–29	166(72.8)	772(72.1)	
≥ 30	44 (19.3)	238 (22.2)	
Missing	2 (<1%)	6 (<1%)	
**Parity**			
0	81 (35.22)	365 (33.89)	0.481
1	72 (31.30)	381 (35.38)	
≥2	77 (33.49)	331(30.73)	
**Gestational weeks**			
<37	90 (39.13)	322 (30)	0.009*
37–40	108(46.96)	622 (57.75)	
>40	32 (13.91)	133 (12.35)	
**Number of gestation**			
Single	228 (99.13)	1050 (97.49)	0.126
Multiple	2(0.87)	27 (2.51)	
**Fetal Presentation**			
Cephalic	229 (99.57)	1038(96.33)	< .01*
Other	1 (0.43)	39 (3.62)	
**Previous bad obstetric history including CS**			
No information	180 (78.26)	528 (49.03)	< .01*
Yes	50 (21.74)	549 (50.97)	
**Uterotonic used before labour**			
No	163(70.87)	1023(95.54)	< .01*
Yes	67(29.13)	49 (4.46)	
**Newborn birth weight (kg)**			
<2.5	28 (12.17)	119 (11.78)	0.62
≥2.5	202 (87.83)	958 (88.95)	
**Childbirth outcome**			
Still birth	12 (5.22)	11 (1.02)	< .01*
Live birth	218 (94.78)	1066 (98.98)	
**Sex of the baby**			
Female	100	449	0.683
Male	119	568	
Missing	11(4.78)	60(5.57)	

*Statistically significant at 95% confidence interval

### Determinants of CS births

The factors influencing the decision for CS birth identified by regression analysis are presented in Table 3. Parity, multiple gestation, non-cephalic presentation, previous bad obstetric history and use of oxytocin for induction or augmentation of labour, were found to significantly influence CS outcome. Results also indicate that with increased parity, the chance of CS decreased significantly. Multiple pregnancy was found to almost quadruple the likelihood of CS (OR: 3.93; 95% CI: 1.25–12.34), while previous history of bad obstetric history including CS birth increased the chance of CS by 35 times. Non-cephalic fetal presentation (breech or oblique lie) was also found to increase the chance of CS birth (OR: 1.34; 95% CI: 4.11–31.31), whereas the use of utero-tonics significantly decreased the likelihood of CS birth by almost 85% (OR: 0.15; 95% CI: 0.09–0.26).

**Table 3:** Determinants of CS births in for-profit private hospitals in urban Bangladesh.

	CS N (%)	AOR	95% CI	p-value
**Mothers' age (Years)**				
≤19	61 (5.7)	1		
20–29	772(72.1)	1.37	0.92–2.03	0.122
≥30	238 (22.2)	1.51	0.87–2.59	0.135
**Mother's parity**				
0	365 (33.89)	1		
1	381 (35.38)	0.47	0.31–0.71	<0.01*
≥2	331(30.73)	0.29	0.17–0.50	<0.01*
**Number of gestation**				
Single	1050 (97.49)	1		
Multiple	27 (2.51)	3.93	1.25–12.34	0.02*
**Gestational age (week)**				
< 37	322 (30)	1		
≥ 37–40	622 (57.75)	1.44	0.89–2.33	0.13
>40	133 (12.35	1.39	0.88–2.19	0.15
**Newborn birth weight (KG)**				
<2.5	119 (11.78)	1		
≥2.5	958 (88.95)	1.34	0.64–2.94	0.42
**Foetal presentation**				
Cephalic	1038(96.33)	1		
Non-cephalic	39 (3.62)	1.34	4.11–31.31	<0.01*
**Bad obstetric history**				
No	528 (49.03)	1		
Yes	549 (50.97)	35.30	13.38–93.10	<0.01*
**Utero tonic used**				
No	1023(95.54)	1		
Yes	49 (4.46)	0.15	0.09–0.26	<0.01*
**Log likelihood -**470.45125 **R**^**2**^ 0.2204 **observation used =** 1299				

AOR: Adjusted Odd Ratio CI: Confidence Interval

*Statistically significant

## Discussion

The high CS rate observed in this analysis corresponds to evidence from other studies in Bangladesh and the surrounding region. For example, a multicounty study done in Asian context found a facility-based CS rate in private and charitable health facilities of 73% and 30% in Bangladesh and Nepal respectively[26]. Another study reported much higher CS rates in private for-profit hospitals compared to public hospitals: greater by 36% in India, 48% in Indonesia and 130% in Bangladesh[27]. Similar patterns are also apparent in developed country contexts such as Switzerland, where the CS rate was 41% in private clinics against 30% in public hospitals[28]. Linked to elevated CS rates are concerns about their inappropriate use. Findings that indicate high rates of CS without medical indications in the private sector exemplify this concern[29]. In this context, application of Robson TGCS has the potential to identify the specific obstetric subgroups that disproportionately contribute to high CS rate and that require regular monitoring[30]. Since the WHO endorsement, some countries have already adopted this system in their regional or national level CS monitoring services[7, 10, 23, 31, 32]. However, data supporting its use remains limited in developing country contexts[10, 20].

Regarding the feasibility of implementing Robson TGCS, our study revealed a minimum number of missing values for the five basic obstetric characteristics required for ten group construction. The validity of the data was also reasonable. Moreover, the relative size of each obstetric group was mostly within the expected range. For example, in the literature, the ratio of “Groups three and four” in combination is generally suggested to be greater than the “Group one and two” in together[12]. Accordingly, in our study, the combined size of “Groups three and four” was appropriately higher (24.2%) than the cumulative percentages of “Groups one and two”, (15.4%). Breech comprising “Groups six and seven” are generally expected to be below 4%[12], while in our study it was found as expected, below 2.7%. However, the high rate of preterm pregnancies (23%) found within the study sample did not coincide with findings from other studies [10]. It may be that the high rate of preterm pregnancies is a result of misreporting the last menstrual period or having a first ultrasound scan later in pregnancy resulting in incorrect calculation of gestational age. In future, careful attention should be given when reporting the foetal gestational age. In pregnancies in which there is mismatch of foetal growth in between ultra-sonography and date calculated from last menstrual period, gestational age should be corrected by comparing physical examination and serial sonography. Another opportunity to correct gestational age is estimating cervical length during labor. This technique has also been reported as efficacious in correct gestational age estimation and hence could reduce preterm births [33].

After validation of data quality, group specific CS rates were explored. According to Robson TGCS analysis, the main three contributors of overall CS rate were previous CS, term elective and preterm pregnancy groups. Previous CS was responsible for one quarter of CS in this study which is consistent with previous research [34–36]. However, elective CS is not the recommended evidence-based practice for the previous CS group, but rather watchful follow-up for spontaneous labor[19, 37]. As an additional precaution during vaginal birth after previous CS (VBACS), it is also recommended that limited use of uterotronic drugs for induction or augmentation should occur. Instead physiological labor induction through amniotomy has proven beneficial in assuring VBACS success[23].

Meta-analysis findings in the broader literature suggest that induction or augmentation of labour during the first stage of labour can reduce unnecessary CS among “Robson one & two” [38]. In our study, logistic regression results similarly suggest that use of oxytocin for the purposes of induction or augmentation of labour could reduce CS rate up to 85%. However, use of such drugs was very low in our sample, with the result that CS rates were high in “Robson one & two” term elective groups. Virtually all CSs were attributed to pre-labour CS in these groups.

High rates of preterm CS observed in this study correspond with studies in the USA where preterm CS rates were over 50%[39]. However, countries with limited technical support to manage preterm low birth weight babies should be cautious in planning the time of delivery. Deferring the birth until term pregnancy with close follow-up may decrease the chance of intensive care unit admission among newborns[33].

The two other obstetric groups,”Robson seven & eight”- multiparous breech and multiple pregnancies—also represent high group-specific CS rates although the relative size of these groups was comparatively small. Having multiple pregnancies, and especially twins, increased the likelihood of delivery by CS by four times. Similarly, non-cephalic presentation (the majority of which were breech presentation) was 1.34 times more likely to be delivered by CS. However, neither multiple pregnancy nor breech presentation were recommended as independent risk factors for CS births in international guideline. Some other pregnancy factors like presentation of first baby, type of twin and mother’s parity were being asked to assess before selecting mode of delivery in these circumstances [40, 41]However, studies comparing planned CS versus vaginal births for twin and breech pregnancies do not report increased neonatal mortalities or morbidities without other concomitant risk factors[40, 42–44].

In our study we also looked at the influence of hospital size on the variation of CS rate in different obstetric sub-groups. It was observed that CS rates in high risk obstetric groups were greater in larger hospitals. For instance, we found that women with transverse or oblique lie babies were presenting mostly in large medical college hospitals. Similarly, the CS rate for abnormal lies in the “Robson nine” group, was nil in small-sized hospitals. The preterm CS rate was also much higher in large hospitals: 44% compared to 24–29% in small to medium -sized hospitals. Since medical college hospitals are better equipped to provide supportive care to preterm and low birth weight babies, it follows greater CSs there. The observed effect of the hospital bed size on CS rate is consistent with other studies that classify this tendency as the “supply-driven model”, whereby the greater the capacity of the health system to offer surgical obstetric care, the higher numbers that are delivering surgically[31].

The most common CS indications reported in this private for-profit hospital study—previous CS, prolonged labor and fetal distress—are similar to findings gathered in public hospital settings[15]. However, according to international recommendations of the National Institute of Excellence (NICE)[37], none of these indications are mentioned as candidates for CS. Indeed, with appropriate and timely intervention, many such cases can be managed successfully by normal delivery[45]. To ensure the best clinical practice, therefore, standard labor monitoring guidelines are an essential first step[12].

### Strengths and limitations

This study included a range of hospital types, from small to medium-sized hospitals, to large medical colleges with the intention of understanding variations between them. By adding cluster effect during logistic regression, we were able to minimize possible biases from obstetric case mixes among similar type of hospitals. A limitation of our study that could not be overcome was a lack of data to validate reported indications of CS. In the large majority of cases, neither partograph sheet nor data indicating the start time of labour pains were available. Thus, we cannot infer whether labour inducing or augmenting medicine was used according to need. Similar to that, to define preterm group we have to rely on reported gestational age. Moreover, the wide confidence interval around certain variables may be a consequence of small numbers of observations, and signal the need for cautious interpretation and generalization.

### Conclusion

The caesarean section (CS) rate in this study of for-profit private health facilities in urban Bangladesh is alarmingly high. The Robson TGCS was found to be a feasible and useful tool for identifying the obstetric groups of women contributing to elevated CS rates. The obstetric sub-groups of women having highest CS rate were elective groups comprising Robson five (previous CS), Robson 10 (preterm) and “Robson two & four” combined (elective term). However, none of these three groups are recommended candidates for CS according to international clinical guidelines. Of additional concern are high CS rates reported in nulliparous women. This increases the risk of repeat CS in the subsequent pregnancy since vaginal birth after previous CS is not a regular practice in this study population. Using Robson TGCS, this first ever study of CS in private for-profit health hospitals in urban Bangladesh precisely identifies the unnecessary burden of CS and where to intervene. The next crucial step would be to engage the multiple level stakeholders to plan effective strategies for safe reduction of CS.
